# Looking for Chinks in the Armor of Bacterial Biofilms

**DOI:** 10.1371/journal.pbio.0050307

**Published:** 2007-11-13

**Authors:** Don Monroe

## Abstract

Researchers are exploring various ways to control the stubborn biofilms that bacteria form on medical devices and in many persistent infections.

David Davies stood at the hospital bedside of his great-aunt, who had recently had all her toes amputated to try to prevent persistent wounds from spreading. It didn't work; doctors would later amputate both of her feet, and she never returned to her independent life. As Davies thinks back to this episode from his high-school days, “I remember wondering ‘How come, in this era of antibiotics, was it not possible to treat what was obviously, to me, an infection?’”

Even today, such nonhealing wounds are common in people with late-stage diabetes like Davies' great-aunt, who have poor circulation, as well as in people with compromised immune systems. Davies, now an associate professor at Binghamton University, says that many doctors treat these debilitating wounds as a problem with the patient, rather than a sign of infection. “There's no excuse for it,” he says.

Wounds are just one example of the huge impact of bacterial biofilms. The United States National Institutes of Health says that 80% of chronic infections are biofilm related. Unlike the more familiar “planktonic” lifestyle, in which bacteria float or swim freely, in biofilms they surround themselves with a complex polymeric matrix, better known as “slime.” As it grows thicker, the film often includes many bacterial species and the matrix develops a complex structure. Traditional antibiotics are often ineffective. “We thought we had it all figured out,” Davies says, but the past 20 years have shown that researchers are still in the “dark ages” when it comes to understanding and controlling bacteria.

As biofilms, bacteria routinely foul industrial equipment and medical devices like catheters and implants, where they form dense layers that cling tightly to the artificial surfaces. They also occur naturally within us, most familiarly as dental plaque. In addition, biofilms are increasingly blamed for recurrent and chronic infections. The case is clear for the Pseudomonas aeruginosa lung infections that haunt cystic fibrosis patients and for recurrent middle ear infections of Haemophilus influenzae. But biofilms are also prime suspects in a long list of other “itises,” including endocarditis, prostatitis, and conjunctivitis.

Researchers have learned much in recent years about the mechanisms that let bacteria establish a beachhead on a surface and work together to form a highly structured matrix that nourishes and protects them. The films are easily seen by electron microscopy on foreign surfaces like catheters. However, definitively establishing a role for biofilms in a particular disease—or in chronic wounds like those of Davies' great-aunt—is difficult, in part because traditional culture and assay techniques work best for planktonic forms. Even as the evidence comes in, however, many researchers are actively seeking unique vulnerabilities of the biofilm state, hoping to control these stubborn films wherever they occur.

## A Stubborn Foe

The usual defenses often fail against bacteria that have formed biofilms. The slimy matrix protects bacteria from assaults such as those by immune cells. In addition, bacteria in a biofilm are 10–1,000 times more resistant to antibiotics than in their planktonic form.

“There's no simple explanation why biofilms are more resistant to antibiotics,” says George O'Toole of Dartmouth. He suggests that resistance is a natural part of adaptation to life on a surface. Although people like to think that we invented antibiotics to disrupt microbial processes, most were adapted from naturally occurring chemicals that the bacteria have evolved to resist. “Almost every antibiotic that's out there is derived from another microbe,” O'Toole says. “Bacteria and fungi have been dealing with this biological warfare for millions or billions of years.”

At first, researchers attributed the resistance mainly to the complex slime that the bacteria secrete. The slime does present a barrier to immune cells like phagocytes. However, experiments show that many antibiotics, as well as nutrients and waste products, readily diffuse through the water-rich matrix.

The slow metabolism of biofilm cells also contributes to their resistance. The bacteria in the film are relatively quiescent and divide only rarely. Antibiotics such as the penicillins, which need to be incorporated in the cell wall, are only effective against actively dividing cells. However, other antibiotics work just as well against quiescent cells, because they target basic cellular processes such as metabolism or protein or DNA synthesis. For reasons that are still being clarified, even these antibiotics are less effective against biofilms.

## O, Pioneers!

**Figure 1:**
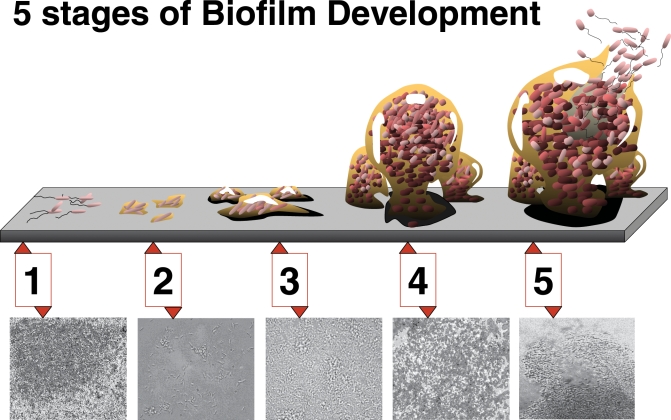
Biofilm Maturation Is a Complex Developmental Process Involving Five Stages Stage 1, initial attachment; stage 2, irreversible attachment; stage 3, maturation I; stage 4, maturation II; stage 5, dispersion. Each stage of development in the diagram is paired with a photomicrograph of a developing P. aeruginosa biofilm. All photomicrographs are shown to same scale. (Image Credit: D. Davis)

Because biofilms, once formed, are so hard to eradicate, many researchers—and makers of medical devices—have tried to stop them from forming in the first place. A biofilm starts when a few pioneer cells use specialized chemical hooks to adhere to a surface. These pioneers help to make a target surface more attractive to subsequent cells, which eventually mature into a complex, structured film (Figure 1).

Makers of medical devices, such as catheters, frequently try to prepare the device surfaces to disrupt the initial adhesion. For example, they may alter the physical characteristics or chemical properties of the surface, such as its hydrophobicity, to make it harder for these first pioneers to stick. “Overall, these approaches haven't been very successful,” asserts Phil Stewart. Stewart heads the Center for Biofilm Research at Montana State University in Bozeman. One reason for this lack of success is that surfaces immersed in bodily fluids develop a coating of bio-friendly material. Once the original surface is even partially covered, bacteria have a place to stick.

## Other Approaches

Some researchers are going beyond simple surface treatments to incorporate biologically active agents into the surfaces of medical devices. Some naturally occurring proteins, like lactoferrin, interfere with bacterial adhesion. In addition, surfaces impregnated with antibiotics can delay biofilm growth, although this technique has the important downside of encouraging resistant bacteria. These surface treatments are directly useful only for human-made devices, but they also provide a testing ground for possible treatments for medical biofilms.

## Strength in Diversity

Researchers are also exploiting the unique biochemistry of bacteria in biofilms, such as a lack of iron or oxygen. Once a biofilm is established, however, it develops a complex structure in which different cells occupy distinct environments. This diversity, both physiological and genetic, is an important part of biofilms' stubbornness, say Pradeep Singh of the University of Washington. First, “the cells are experiencing different environmental conditions, so their physiology is by nature different. The guys on the outside have very different physiology from the guys on the inside, so if you have a treatment or a target or a drug that affects one, the other may not be affected.” Levels of acidity, oxygen, and iron, for example, vary widely through the film.

As the cells grow in these varying environments, they diverge genetically as well. In one study of biofilm growth, Singh says, “if we started with genetically identical population of cells, after five or ten days we'd find that population had actually diversified and was more like an old-growth forest than an monoculture of cells.” Like the forest, Singh suggests, the diversity of the biofilm could be a key element of its robust response to antibiotics and other assaults.

The diversity clearly lets biofilms recover rapidly. Researchers speak of a small population of cells, called “persisters,” that for one reason or another survive an immune or antibiotic attack. Afterwards, says Bill Costerton of the University of Southern California, “if you are a persister, you wake up in a puree of the guts of your neighbors that contains every molecule you ever needed.” These well-fed survivors can rapidly reestablish the film once the assault is over, he says. “Biofilms have a regrowth rate that is truly phenomenal.”

Dealing effectively with this diversity may require a shift from the traditional single-antibiotic approach to bacteria. “If you look at a lot of other treatment regimens for other diseases—cancer, HIV—it's almost always a cocktail of drugs targeting different aspects of the disease process,” says Dartmouth's O'Toole. Combining antibiotics with other compounds that disrupt the formation or survival of the film might render the bacteria more susceptible to antibiotics—and could reduce antibiotic resistance, as well.

## Majority Vote

As bacteria arrange into a biofilm, the expression of scores of genes increases or decreases compared to their level in the planktonic form, raising hopes for targeting the biofilm-related pathways. Although many of the changes are still not understood, those involved in interbacterial signaling are especially promising. Indeed, for several species, researchers have identified signaling chemicals that perform in “quorum sensing,” which induces an abrupt change in phenotype when the density of nearby cells (and thus the signaling concentration) exceed a threshold level.

At first, quorum sensing seemed to be a critical pathway for forming biofilms, although the precise pathways differ somewhat between species. Recent results, however, show that mutant bacteria that can't do quorum sensing can sometimes form biofilms nonetheless. “The whole quorum sensing thing is still kind of shaking out,” says Montana State's Stewart. Although he suspects that disrupting quorum sensing will remain an important tool, Stewart says, “my read at this point is it's not as simple as ‘they have to be able to communicate to build a biofilm.’”

In addition, Washington's Singh says that the bacteria from the lungs of cystic fibrosis patients who have longstanding biofilm infections are often mutants that can no longer perform quorum sensing. He suspects that once a biofilm is established, the chemical signals could serve as a beacon for the immune system. In their ongoing battle to evade detection, the bacteria may evolve to suppress quorum sensing when it is not helpful.

## Not Just a Pretty Phage

**Figure 2:**
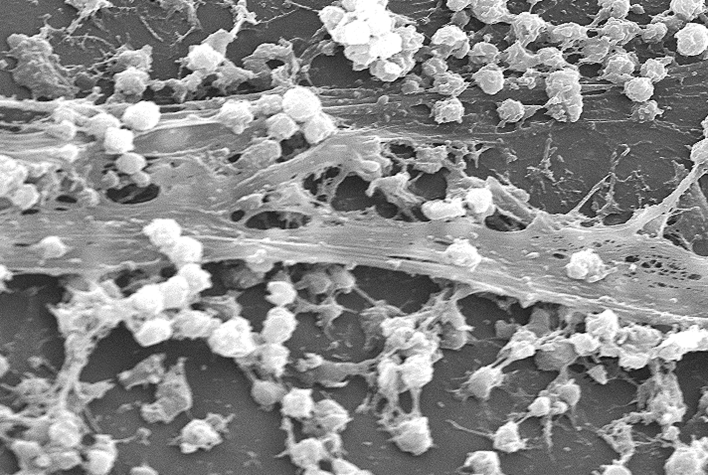
A S. aureus Biofilm on the Surface of a Medical Catheter That Was Removed from a Patient The round bacteria secrete a complex “slime” that helps protect them from attack by antibiotics and other antimicrobial agents. Electron micrograph magnified 2363×. (Image Credit: CDC/Rodney M. Donlan, Ph.D.; Janice Carr (PHIL #7488), 2005)

Once biofilms develop, their most obvious distinguishing feature is the slime they secrete, which both holds the cells together and helps protect them (Figure 2). This richly structured matrix consist of a goulash of polysaccharides, as well as lipids, proteins, and even nucleic acids. Chemicals that attack this slime chemically can disrupt a variety of biofilms on medical equipment. Such a general assault is tricky for internal infections, but can be effective when applied directly to wounds.

Another strategy exploits viruses called phages that target bacteria. Phages tend to be highly specific, targeting particular species and even strains of bacteria. This specificity can be useful, especially for systemic treatments, because it can avoid disrupting natural, beneficial populations of bacteria, such as those in the gut. These beneficial bacteria often occur in biofilms themselves, and can naturally suppress more troublesome strains.

However, doctors might need to know which specific organisms causing an infection in order to choose a phage to treat it. This may not be as difficult as it seems, since a few organisms (such as P. aeruginosa, Escherichia coli, Staphylococcus aureus, and S. epidermidis) are responsible for a large fraction of infections. Researchers in the former Soviet Union have used phage to treat infections for decades and have developed cocktails that attack a spectrum of organisms. In this country, there are both practical and regulatory challenges to introducing active viruses into patients, but that may be changing.

“My sense is that increasingly the US community is open to the potential of using phage,” says Jim Collins of Boston University. With colleague Timothy Lu, Collins has used synthetic biology to alter natural viruses, adding genes for enzymes that attack the slime. The phage first hijack the bacterial machinery to replicate themselves, then break the cells open to release not just the copies, but the enzyme. Compared to the phage alone, this engineered phage “was about 100 times more effective” at disrupting a laboratory biofilm, Collins says.

Rodney Donlon heads a team at the US Centers for Disease Control and Prevention aiming to reduce infections in medical devices. He says that the engineered phage is “very interesting,” although the genetic engineering of the virus could raise further concerns about its release into the environment. Still, Donlon emphasizes that phage naturally only attack bacteria. “They will not infect human or plant cells.” But the phage are not without problems, such as the release of toxins when the cells split open or immune responses to the viruses like those that have dogged gene therapy trials.

Donlon has explored phage for devices, incorporating them into coatings on catheters. In what Donlon describes as a “proof of principle,” these coatings suppress the growth of biofilms in laboratory tests. Interestingly, they appear to remain active even after exposure to bodily fluids.

## Harnessing Dispersion

Since each biofilm is different, however, researchers may need to tailor their approach to a particular species and matrix—or a combination. To overcome this problem, Binghamton's Davies looked at the final stage of the life cycle of biofilms: autodispersal. In this stage, regions of the film spontaneously disperse as cells dissolve the matrix by secreting enzymes and revert to their planktonic form.

Davies' team found a signaling molecule that initiates autodispersal. As in quorum sensing, this signal induces a profoundly different behavior above a threshold concentration. The chemical signals are completely different, however, and instead of causing films to coalesce, it causes them to break up.

One of the most tantalizing aspects of this molecule is that it appears to be universal across bacterial species. Although the enzymes required to degrade the matrix differ between species, the same signaling molecule triggers the process. Davies' team has identified the molecule and verified that a synthesized version induces dispersal even in films that are below the threshold density, and works even in multispecies biofilms.

For widespread infections like those in cystic fibrosis, instantaneously releasing billions of bacteria in their planktonic form could cause even worse problems than the film. But for localized infections, like those in the sinus or middle ear, Davies thinks his dispersion-inducer could make intractable infections vulnerable to traditional antibiotics, or even to normal immune response. “Most infections are very localized, and it's not easy for the cells to really get very far from the site of the biofilm infection,” he says. At a more personal level, Davies hopes that the dispersal agent could help millions of people with nonhealing wounds, like those of his aunt.

## Dealing with Chronic Wounds

In fact, recent results show biofilms in many chronic wounds, says Randall Wolcott, who heads the Southwest Regional Wound Care Center in Lubbock, Texas. “Six months from now, I think the wound-care community will fully accept biofilm as a major barrier to healing.”

Although worried about being branded as “one of those alternative-medicine types,” Wolcott has been exploring anti-biofilm therapies. “I watched so many people die, in their forties and fifties, a piece at a time,” he says. “I'd just had enough.” Overall, he says, “biofilm disease kills more people than cancer.”

For wounds that don't respond to standard-of-care wound treatment, including mechanical removal of damaged tissue, Wolcott adds as many as six or seven agents to kill bacteria and disrupt the biofilms. He also uses phages, which qualify as natural substances and has occasionally seen “wounds that have been present for years go on to heal up in weeks.”

These ongoing studies show the potential for treatments directed at biofilms, especially in combinations that counter the natural diversity of the biofilm populations. “We know how to manage biofilms,” says Wolcott. “We just need to bring it into medicine.”

